# The use of S100 proteins testing in juvenile idiopathic arthritis and autoinflammatory diseases in a pediatric clinical setting: a retrospective analysis

**DOI:** 10.1186/s12969-020-0398-2

**Published:** 2020-01-16

**Authors:** Najla Aljaberi, Elena Tronconi, Grant Schulert, Alexei A. Grom, Daniel J. Lovell, Jennifer L. Huggins, Michael Henrickson, Hermine I. Brunner

**Affiliations:** 1 Division of Rheumatology, Cincinnati Children’s Hospital Medical Center, 3333 Burnet Avenue, Cincinnati, OH 45229 USA; 2 Pediatric Unit, Department of Medical and Surgical Sciences, University of Bologna Hospital of Bologna Sant’Orsola-Malpighi Polyclinic, Bologna, Emilia-Romagna Italy

**Keywords:** S100 proteins, Biomarkers, Juvenile idiopathic arthritis, Autoinflammatory disease, Periodic fever syndrome

## Abstract

**Background:**

Serum phagocyte-derived alarmins S100A8/9 and S100A12 are considered useful for the assessment of inflammatory diseases. Our study evaluated the use of S100 proteins in a pediatric clinical setting for estimating disease activity and supporting diagnosis.

**Methods:**

Patients (*n* = 136) who had S100 proteins tested as part of clinical care were included in this study and relevant information obtained from the medical record: C-reactive protein (CRP), disease activity status (inactive: = 0 joint; active: > 0 active joint), systemic symptoms in systemic JIA (sJIA), and symptoms of flare of other autoinflammatory and fever syndromes. Patients were categorized as: sJIA, non-systemic JIA (nsJIA), other defined autoinflammatory syndromes (AID) and systemic undifferentiated recurring fever syndromes (SURFS).

**Results:**

Patients with sJIA (*n* = 21) had significantly higher levels of S100A8/9 and S100A12 compared to patients with nsJIA (*n* = 49), other AIDs (*n* = 8) or SURFS (*n* = 14) (all *p* < 0.0001). Compared to CRP [area under the receiver operating characteristics curve (AUC) = 0.7], S100 proteins were superior in differentiating sJIA from AID and SURFS [AUC = 0.9]. S100A8/9 and S100A12 levels were not associated with disease activity in nsJIA, AID or SURFS. S100A8/9 and S100A12 levels were significantly higher in active sJIA compared to inactive (*p* = 0.0002 and *p* = 0.0002 respectively).

**Conclusion:**

Compared to other autoinflammatory and fever syndromes, sJIA patients have markedly higher levels of S100A8/9 and S100A12 proteins which may assist with diagnosis. S100 levels slightly outperformed CRP in distinguishing sJIA from other diagnoses and in sJIA disease activity. S100 proteins may aid in monitoring disease activity in sJIA patients.

## Background

The S100 proteins are the largest subgroup within the family of Ca2 + −binding proteins characterized by the helix-loop-helix (EF-hand) structural motif [1]. Among others, these proteins are involved in cell signal transduction, cell differentiation, regulation of cell motility, transcription and cell cycle progression. Apart from these intracellular functions, some S100 proteins can be secreted from cells and exhibit cytokine-like extracellular functions [2]. Two highly studied proteins are the heterodimer S100A8/A9 (also: MRP8/14 or calprotectin) and the S100A12 protein (also: calgranulin C). Both are predominantly secreted by phagocytes [3]. S100A8/9 and S100A12 can serve as endogenous toll-like receptor 4 agonists and thus activate the innate immune response as so-called alarmins [4]. Serum S100 levels are elevated in patients with autoimmune diseases, and several studies have demonstrated their usefulness as diagnostic markers of inflammation [5, 6]. Levels of S100A8/A9 and S100A12 have been considered sensitive biomarkers of disease activity in rheumatologic disorders, and may be even more reflective of the degree of inflammation than erythrocyte sedimentation rate (ESR) and C-reactive protein (CRP) [7]. Prior studies suggested that S100A8/9 levels are associated with disease activity in patients with rheumatoid arthritis [5] and juvenile idiopathic arthritis (JIA) [6]. S100A12 has also been linked to autoimmune diseases, where it was strongly expressed in inflamed tissues from patients with adults with chronic arthritis [8].

The aims of this study were to evaluate the utilization of S100 proteins in the clinical setting as supportive diagnostic tools as well as their performance in evaluating disease activity.

## Materials and methods

### Patients and data

Included in this cross-sectional study were patients in whom serum S100A8/9 and S100A12 levels were measured as part of standard clinical care by providers in the rheumatology clinic at Cincinnati Children’s Hospital between April 2017 and May 2019. For these patients, we extracted from the electronic medical record the following data at the time of S100 measurement: clinical diagnosis, CRP levels, JIA disease activity measures. For systemic JIA (sJIA) and patients with other autoinflammatory syndromes, we also noted whether the disease was clinically active or inactive at the time of S100 measurement. Levels of CRP were documented only if they were within 3 days of S100 protein measurement.

For the purpose of the analysis, patients were grouped based on clinical diagnosis in one of four groups: sJIA, non-systemic forms of JIA (nsJIA), other defined autoinflammatory disease (AID), or systemic undifferentiated recurring fever syndromes (SURFS). JIA patients all fulfilled the International League of Association for Rheumatology Criteria [9]. In the AID category, we included patients with defined genetic or clinically diagnosed fever syndromes other than sJIA. This group is comprised of patients with familial Mediterranean fever (FMF), Tumor Necrosis Factor Receptor Associated Periodic Syndrome, Periodic Fever Aphthous stomatitis Pharyngitis and Adenitis syndrome and Muckle-Wells syndrome. Patients with periodic or recurrent patterns of fever but without a defined disease were categorized as SURFS. For purposes of comparison against sJIA, the AID and SURFS groups were combined occasionally into one group “AID+SURFS”.

For patients diagnosed with JIA the presence of active JIA was defined as an active joint count (AJC) of > 0, irrespective of the subset of JIA (sJIA, nsJIA), with or without the addition of systemic symptoms indicative of flare in sJIA such as fever and rash. Data such as patient and provider global assessment of disease were missing not infrequently hence were omitted from the statistical analysis. Active AID and SURFS were defined by the presence of typical clinical features of flares as documented by the treating pediatric rheumatologist at the time of S100 protein testing. Encounters that lacked clear mention of disease activity were excluded from analyses that addressed the relationship of S100 proteins and the presence of active disease.

This study was approved by the Cincinnati Children’s Institutional Review Board (IRB) (2018–2549).

### Laboratory assessment

The Human S100A12/EN-RAGE ELISA Kit (Medical and Biological Laboratories Co., Japan) and Quantikine Human S100A8/S100A9 Heterodimer Immunoassay (R&D Systems, Minneapolis, MN) utilize the quantitative sandwich enzyme immunoassay technique.

Testing was performed in the CCHMC Diagnostic Immunology Laboratory which participates in the College of American Pathologists (CAP) Laboratory Accreditation Program and has Clinical Laboratory Improvement Amendments (CLIA) certification through the Centers of Medicare and Medicaid Services (CMS). The normal range of S100A8/9 per this assay is 716–3004, while that for S100A12 is 32–385.

### Data analysis

Graphpad Prism Version 7.05 (Graphpad, San Diego, CA) was used for data analyses. Descriptive statistics were calculated for all variables. Between-group comparisons were done by the Mann-Whitney U test and the Kruskal-Wallis test. Spearman’s test was used to study the correlation between S100 proteins, CRP and AJC. Receiver operating characteristic curve (ROC) analysis was done to assess the performance of the S100 proteins in distinguishing patients with different diagnoses in terms of overall sensitivity and specificity as represented by the area under the ROC curve (AUC). AUC values for diagnostic tests can be interpreted as outstanding, excellent, good, and fair, if the AUC values are in the range of 0.91–1.0,

0.81–0.90, 0.71–0.80, and 0.61–0.70, respectively. For values for CRP levels lower than the lower limits of normal (< 0.29 mg/dL) were imputed as a value of 0.1. These values were eliminated from the correlation analysis. *P*-values < 0.05 (2-sided tests) were considered statistically significant.

## Results

### Distribution of S100 proteins among diagnoses

A total of 136 patients were included in this review (92 with active disease, 44 with inactive disease). In the 92 patients with active disease, 89 patients had S100A8/9 collected and 76 had S100A12 collected. The levels of S100A8/9, S100A12 and CRP in the different disease groups are shown in Table 1.

**Table 1:** Levels of Serum S100A8/9, S100A12 and CRP by diagnoses with pediatric rheumatic diseases during active disease

	sJIA *N* = 21	nsJIA *N* = 49	AID *N* = 8	SURFS *N* = 14	*p* value
S100A8/9 (ng/ml)	*N* = 19 31,465 (3708, 47,172)	*N* = 48 1471 (765, 2589)	*N* = 8 1352 (893, 5548)	*N* = 14 2224 (1842, 4175)	< 0.0001
S100A12 (ng/ml)	*N* = 21 2075 (278, 5612)	*N* = 36 100 (55, 218)	*N* = 7 80 (54, 544)	*N* = 12 130 (108, 259)	< 0.0001
CRP (mg/dL)	*N* = 15 2.7 (0.7, 12.6)	*N* = 34 0.1 (0.1, 0.7)	*N* = 6 1.3 (0.1, 4.5)	*N* = 7 0.1 (0.1, 3.5)	0.0016
INACTIVE	*N* = 12	*N* = 23	*N* = 5	*N* = 4	N/A

Comparisons between groups of diagnoses are done by Kruskal-Wallis test. Values shown as median (inter-quantile range). Abbreviations: *nsJIA* non-systemic juvenile idiopathic arthritis, *sJIA* systemic juvenile idiopathic arthritis, *AID* autoinflammatory disease, *SURFS* systemic undifferentiated recurring fever syndromes, *CRP* C-reactive protein. The normal range of S100A8/9 per this assay is 716–3004, while that for S100A12 is 32–385

**Fig. 1:**
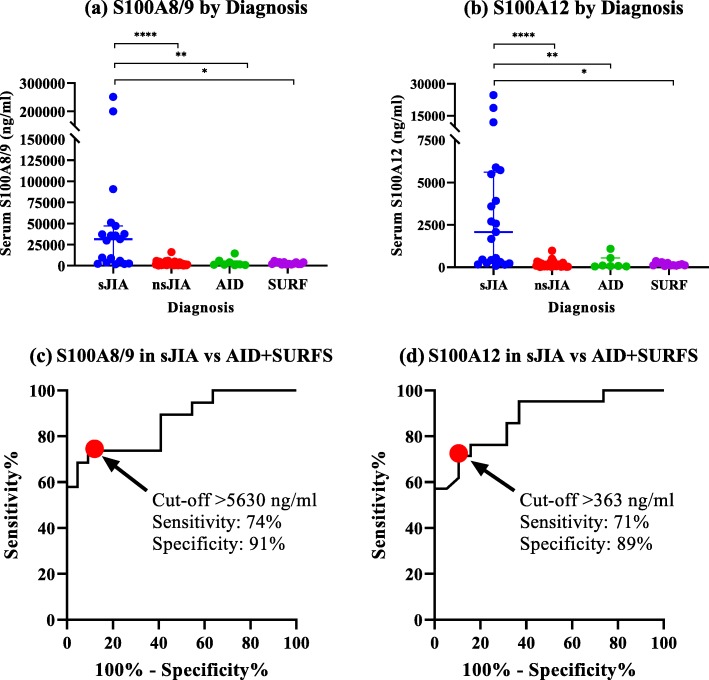
**a** and **b** show serum levels of S100A8/9 (**a**) and S100A12 (**b**) across diagnoses. Values represent median and IQR (error bars). Comparison between diagnoses are done by Kruskal-Wallis test. **c** and **d** show receiver operating charachteristic (ROC) curves for S100A8/9 (**c**) and S100A12 (**d**) in differentiating systemic juvenile idiopathic arthritis (sJIA) vs. Other autoinflammatory syndromes designated as autoinflammatory disease + systemic undifferentiated recurring fever syndromes (AID + SURFS). The area under the curve (AUC) for both S100A8/9 and S100A12 is 0.9. Sensitivities and specificties for the cut-off values are outlined. * *p* ≤ 0.05, ** *p* ≤ 0.01, *** *p* ≤ 0.001, **** *p* ≤ 0.0001

S100A8/9 level was significantly higher in sJIA compared to all other disease categories (median in ng/ml; 31,465 for sJIA vs. 1471 for nsJIA, 1352 for AID, 2224 for SURFS; *p* < 0.0001). S100A12 levels also were significantly higher with sJIA than all other disease categories (median in ng/ml; 2075 for sJIA vs. 100 for nsJIA, 80 for AID, 130 for SURFS; *p* < 0.0001) (Fig 1a and b). Levels of CRP were also highest in sJIA patients compared to the other categories (*p* 0.002).

We tested the correlation between CRP values (obtained on the same day) and S100 proteins while eliminating values < 0.29 mg/dL. There was a strong correlation between CRP and both S100A8/9 and S100A12 (*r =* 0.9 and *r* = 0.8 respectively; *p* < 0.0001 for both).

We analyzed the performance of the S100 proteins in supporting the diagnosis of sJIA compared to the other autoinflammatory syndromes (AID and SURFS combined into one category; “AID+SURFS”). Both S100A8/9 and S100A12 showed a good ability to differentiate sJIA from other autoinflammatory diseases (AUC = 0.9 for both) compared to AUC = 0.7 for CRP (Fig. 1c). At a cut-off value of 5630 ng/ml, S100A8/9 was 74% sensitive and 91% specific in differentiating between sJIA and AID+SURFS. At a cut-off value of 363 ng/ml, S100A12 was 71% sensitive and 89% specific in differentiating between sJIA and AID+SURFS. Additional cut-off values and their respective coordinates on the ROC curve are shown for S100A8/9 and S100A12 in Additional file 1: Tables S1 and S2 respectively.

### S100 proteins and disease activity

We analyzed the association of S100A8/9 and S100A12 with disease activity in the JIA group. The JIA group consisted of 72 patients with nsJIA and 33 patients with sJIA. In each respective group, patients were divided into active or inactive categories as described previously.

Neither S100A8/9 nor S100A12 were related to disease activity in nsJIA (*p* = > 0.9 and *p* = 0.82, respectively). Levels of CRP were more closely related to nsJIA activity but not statistically significant (*p* = 0.07). S100 proteins and AJC were not correlated with each other in either sJIA or nsJIA patients (data not shown).

Children with active sJIA (*n* = 22, [AJC > 0 and/or systemic symptoms]) had significantly higher S100A8/9 (median in ng/mL; active sJIA vs. inactive sJIA = 31,465 vs. 1685; *p* = 0.0001) and S100A12 levels (median in ng/mL; active sJIA vs. inactive sJIA = 2075 vs. 130; *p* = 0.0002) as compared to sJIA patients without active disease (*n* = 12, [AJC = 0 and/or lack of systemic symptoms]). CRP levels were also statistically higher in patients with active vs. inactive sJIA (*p* = 0.026).

Furthermore, we assessed S100 protein levels during sJIA flares, differentiating whether systemic features were present (*n* = 12) or not (*n* = 7). Levels of S100A8/9 and S100A12 were both significantly higher in patients with systemic sJIA flares compared to articular-only sJIA flares (*p* = 0.004, and *p* = 0.006 respectively). However, the number of patients included in this analysis was small.

We assessed the distribution of S100A8/9 and S100A12 with regard to disease activity in the AIDS+SURFS group. Similarly, patients included in this analysis were those with clear documentation of presence of flare symptoms consistent with their diagnosis. We found no association between S100A8/9 and S100A12 and disease activity (*p* = 0.945, and *p* = 0.885 respectively). Levels of CRP were not indicative of disease activity either (*p* = 0.6).

Due to small numbers of distinct genetic AIDs, further analysis to compare those diseases was not possible. In particular regarding FMF, we had 3 patients, 2 of which had heterozygous MEFV gene mutations and 1 had no detectable MEFV mutations. Despite reported clinical activity in those patients, S100A12 levels were not remarkably elevated (median in ng/ml [inter-quantile range]: 80 [54, 1082]).

## Discussion

The usefulness of phagocyte-specific proteins of S100A8/9 and S100A12 as markers of inflammation has been demonstrated in several reports. In the present study we reviewed the utilization of these proteins as biomarkers in a clinical setting for the management of individual patients. We found that both S100A8/9 and S100A12 are particularly elevated in sJIA compared to nsJIA and other autoinflammatory diseases. Further, we report that S100 proteins can be used to discriminate sJIA from AID and SURFS. The results of our study are in line with other reports outlining the ability of S100A8/9 to distinguish sJIA from other entities, some of which include febrile clinical presentations (periodic fevers and systemic infections) [7]. Wittkowski et al. [10] reported that serum concentrations of S100A12 were highly elevated in sJIA and FMF but not with other fever syndromes. In our very small number of FMF patients (2/3 with heterozygous MEFV mutations) with clinically active disease, this was not observed. In our heterogeneous group of AID patients, S100A8/9, S100A12 and CRP were not helpful in discriminating active from inactive disease states. Nonetheless, use of S100 proteins and in particular S100A12 in monitoring disease activity has been reported to be helpful in FMF in line with what has been reported in the past in larger prospective studies [11].

There are several reports suggesting an association between S100 proteins and JIA activity, MD global and disability as measured by the Childhood Health Assessment Questionnaire (CHAQ) [6, 12]. Our study did not show an association between S100 proteins and articular disease activity in patients with JIA. This finding aligns with lack of S100 proteins ability in predicting flares in non-systemic polyarticular JIA patients per a recent report from our group [13]. However, in the sJIA subset, the levels of S100 proteins were associated with disease activity for systemic symptoms (fever and rash), indicating the potential utility of S100 proteins in monitoring disease course in this subset of patients. Using the JIA core set criteria in defining active and inactive disease, Holzinger et al. found S100A8/9 levels to be predictive of flare in sJIA patients, and that a cut-off of 740 ng/ml was 92% sensitive and 88% specific in diagnosing a flare in sJIA patients [14]. Despite the lack of prospective data in our cohort to study flare prediction in sJIA, our results show the association between S100 proteins and disease activity. We suspect that monitoring of S100 proteins in sJIA patients is likely of high value, among other markers that are currently studies such as interleukin 18 (IL-18), ferritin and other cellular markers [4].

Our study is limited by the cross-sectional nature and the small number of patients in some subgroups. Further work to outline the importance of S100A8/9 and S100A12 requires a larger number of patients and prospective documentation of validated disease activity measures. Additional investigation of these markers individually or in combination with conventional measures could reveal their contribution to clinical decision making. In addition, focused work on the effect of different biologic medications, in particular interleukin-6 blocking biologics, on the expression of these proteins can be of great benefit to clinical practice.

## Conclusion

The results of this cross-sectional analysis demonstrate that S100A8/9 and S100A12 proteins are markedly elevated in sJIA compared to nsJIA and other autoinflammatory diseases. The S100 proteins are useful in distinguishing sJIA from other autoinflammatory and fever syndromes with high sensitivity and specificity. Levels of S100A8/9 and S100A12 are an indicators of disease activity in sJIA but not in other autoinflammatory syndromes or nsJIA.

## Supplementary information

**Additional file 1: Table S1**. Cut-off values of S100A8/9 for the differentiation between sJIA and other autoinflammatory and fever syndrome patients (AIDS+SURFS) and their coordinates on ROC analysis. **Table S2**. Cut-off values of S100A12 for the differentiation between sJIA and other autoinflammatory and fever syndrome patients (AIDS+SURFS) and their coordinates on ROC analysis. **Table S3**. Demographics and clinical characteristics of patients with AIDs*.

## Data Availability

The datasets used and/or analyzed during the current study are available from the corresponding author on reasonable request.

## Abbreviations

AID: Autoinflammatory disease
AJC: Active joint count
CHAQ: Childhood Health Assessment Questionnaire
FMF: Familial Mediterranean fever
JIA: Juvenile idiopathic arthritis
nsJIA: Non-systemic juvenile idiopathic arthritis
ROC: Receiver operating characteristic
sJIA: systemic juvenile idiopathic arthritis
SURFS: Systemic undifferentiated recurring fever syndrome
