# Supplementary material for: The use of S100 proteins testing in juvenile idiopathic arthritis and autoinflammatory diseases in a pediatric clinical setting: a retrospective analysis
Source: Pediatr Rheumatol Online J. 2020 Jan 16;18:7. doi: 10.1186/s12969-020-0398-2 (PMC6966841; doi:10.1186/s12969-020-0398-2)
Supplement: Supplementary file 1 — Additional file 1: Table S1. Cut-off values of S100A8/9 for the differentiation between sJIA and other autoinflammatory and fever syndrome patients (AIDS+SURFS) and their coordinates on ROC analysis. Table S2. Cut-off values of S100A12 for the differentiation between sJIA and other autoinflammatory and fever syndrome patients (AIDS+SURFS) and their coordinates on ROC analysis. Table S3. Demographics and clinical characteristics of patients with AIDs*. [file 12969_2020_398_MOESM1_ESM.docx]

Supplementary Table 1. Cut-off values of S100A8/9 for the differentiation between sJIA and other autoinflammatory and fever syndrome patients (AIDS+SURFS) and their coordinates on ROC analysis.

| S100A8/9  Cut-off value (ng/ml) | Sensitivity (95% CI) | Specificity (95% CI) | +LR (95% CI) | -LR (95% CI) |
| --- | --- | --- | --- | --- |
| ≥702 | 100 (82-100) | 0 (0-15) | 1.0 (1.0-1.0) | - |
| >1463 | 100 (82-100) | 36 (17-59) | 1.6 (1.1-2.2) | 0.0 (-) |
| >1636 | 95 (74-100) | 36 (17-59) | 1.5 (1.1-2.1) | 0.1 (0.0-1.1) |
| >2038 | 95 (74-100) | 45 (24-68) | 1.7 (1.2-2.6) | 0.1 (0.0-0.8) |
| >2104 | 89 (67-99) | 45 (24-68) | 1.6 (1.1-2.5) | 0.2 (0.1-1.0) |
| >3708 | 74 (49-91) | 59 (36-79) | 1.8 (1.0-3.2) | 0.5 (0.2-1.0) |
| >5630^+^ | 74 (49-91) | 91 (71-99) | 8.1 (2.1-31.2) | 0.3 (0.1-0.6) |
| >5893 | 68 (43-87) | 91 (71-99) | 7.5 (1.9-29.2) | 0.4 (0.2-0.7) |
| >6050 | 68 (43-87) | 95 (77-100) | 15.1 (2.2-104.7) | 0.3 (0.2-0.6) |
| >9606 | 58 (34-80) | 95 (77-100) | 12.7 (1.8-89.8) | 0.4 (0.3-0.8) |
| >14542 | 58 (34-80) | 100 (85-100) | - | 0.4 (0.2-0.7) |
| >250906 | 0 (0-18) | 100 (85-100) | - | 1.0 (1.0-1.0) |

^+^ Suggested cut-off value for optimal sensitivity and specificity.

Abbreviations: sJIA: systemic juvenile idiopathic arthritis; AID: autoinflammatory disease; SURFS: systemic undifferentiated recurring fever syndromes, CI: confidence interval; LR: likelihood ratio.

Supplementary Table 2. Cut-off values of S100A12 for the differentiation between sJIA and other autoinflammatory and fever syndrome patients (AIDS+SURFS) and their coordinates on ROC analysis.

| S100A12  Cut-off value (ng/ml) | Sensitivity (95% CI) | Specificity (95% CI) | +LR (95% CI) | -LR (95% CI) |
| --- | --- | --- | --- | --- |
| ≥47 | 100 (84-100) | 0 (0-18) | 1.0 (1.0-1.0) | - |
| >81 | 100 (84-100) | 26 (9-51) | 1.4 (1.0-1.8) | 0.0 |
| >89 | 95 (76-100) | 26 (9-51) | 1.3 (1.0-1.7) | 0.2 (0.0-1.4) |
| >140 | 95 (76-100) | 63 (38-84) | 2.3 (1.3-4.3) | 0.2 (0.1-0.7) |
| >167 | 86 (64-97) | 63 (38-84) | 2.2 (1.3-3.7) | 0.2 (0.1-0.8) |
| >240 | 76 (53-92) | 68 (43-87) | 2.4 (1.2-4.9) | 0.4 (0.2-0.8) |
| >299 | 76 (53-92) | 84 (60-97) | 4.8 (1.7-14) | 0.3 (0.1-0.6) |
| >315 | 71 (48-89) | 84 (60-97) | 4.5 (1.5-13.2) | 0.3 (0.2-0.7) |
| >363^+^ | 71 (48-89) | 89 (67-99) | 6.8 (1.8-25.9) | 0.3 (0.2-0.6) |
| >459 | 62 (38-82) | 89 (67-99) | 5.9 (1.5-22.8) | 0.4 (0.2-0.8) |
| >544 | 57 (34-78) | 95 (74-100) | 10.9 (1.6-75.8) | 0.5 (0.3-0.7) |
| >1082 | 57 (34-78) | 100 (82-100) | - | 0.4 (0.3-0.7) |
| >24690 | 0 (0-16) | 100 (82-100) | - | 1.0 (1.0-1.0) |

^+^ Suggested cut-off value for optimal sensitivity and specificity.

Abbreviations: sJIA: systemic juvenile idiopathic arthritis; AID: autoinflammatory disease; SURFS: systemic undifferentiated recurring fever syndromes, CI: confidence interval; LR: likelihood ratio.

Supplementary table 3. Demographics and clinical characteristics of patients with AIDs*.

|  | Disease activity | S100A8/9 (ng/ml) | S100A12 (ng/ml) | CRP (mg/dL) | Genetic testing results |
| --- | --- | --- | --- | --- | --- |
| FMF (n=4)  patient 1  patient 2  patient 3  Patient 4 | Active  Active  Active  Inactive | 1240  702  14542  1707 | 80  54  1082  146 | <0.29  -  6.5  - | Heterozygous variant V726A in MEFV gene  Homozygous variant E148Q in MEFV gene  Heterozygous variant K695R in MEFV gene  Negative genetic testing |
| TRAPS (N=1)  patient 1 | Active | 6050 | 544 | - | Negative genetic testing |
| MWS (n=1)  patient 1 | Active | 1463 | 101 | 2.51 | Heterozygous variant R327Q in CIAS1 gene |
| PFAPA (n=8)  patient 1  patient 2  patient 3Δ  patient 4  patient 5  patient 6  patient 7  patient 8 | Inactive  Active  Unknown  Active  Active  Inactive  Inactive  Inactive | 7312  4043  470  1147  808  946  -  982 | 494  -  24  65  47  73  102  66 | -  3.85  <0.29  <0.29  <0.29  -  -  <0.4 | -  -  -  -  -  -  -  - |

* This includes patients with inactive disease or unknown disease activity status.

Δ This patient was not included in the analysis since the disease activity status is not known at the time of S100 protein testing.

Abbreviations: AID: autoinflammatory disease; FMF: familial Mediterranean fever; TRAPS: TNF-receptor associated periodic syndrome; MWS: Muckle-Wells syndrome; PFAPA: Periodic Fever, Aphthous Stomatitis, Pharyngitis, Adenitis; CRP: C-reactive protein. The normal range of S100A8/9 per this assay is 716-3004, while that for S100A12 is 32-385.
